# Acute Lymphocytic Leukemia with Bilateral Renal Masses Masquerading as Nephroblastomatosis

**DOI:** 10.1155/2015/806494

**Published:** 2015-11-03

**Authors:** Poonam Thakore, Salim Aljabari, Curtis Turner, Tetyana L. Vasylyeva

**Affiliations:** Department of Pediatrics, Texas Tech University Health Sciences Center School of Medicine, Amarillo, TX 79106, USA

## Abstract

Acute lymphoblastic leukemia (ALL) is the most common malignancy in the pediatric patient population. However, renal involvement as the primary manifestation of ALL is rare. We report a case of a 4-year-old boy with bilateral renal lesions resembling nephroblastic rests as the first finding of early stage ALL preceding hematological changes and subsequent classic clinical findings by two weeks. These renal hypodensities completely resolved after one week of induction chemotherapy. This case demonstrates that renal involvement can be the only initial presenting finding of leukemia. Children with lesions resembling nephroblastic rests need appropriate surveillance due to the risk of malignant disease.

## 1. Introduction

Acute lymphoblastic leukemia (ALL) is the most common malignancy in children between ages of 2 and 5 years [1]. The initial clinical presentation of ALL is nonspecific and may include fatigue, fever, infection, bone pain, organomegaly, and anemia secondary to infiltration of blast cells in bone marrow, peripheral blood, and extramedullary organs. As the disease progresses, other organs, such as the kidneys, may be affected. However, renal involvement in the early stages of ALL is rarely seen [2]. This report details the case of a child with bilateral renal lesions resembling nephroblastic rests as the first finding of ALL preceding the development of the classic features of ALL.

## 2. Case Report

**Figure 1:**
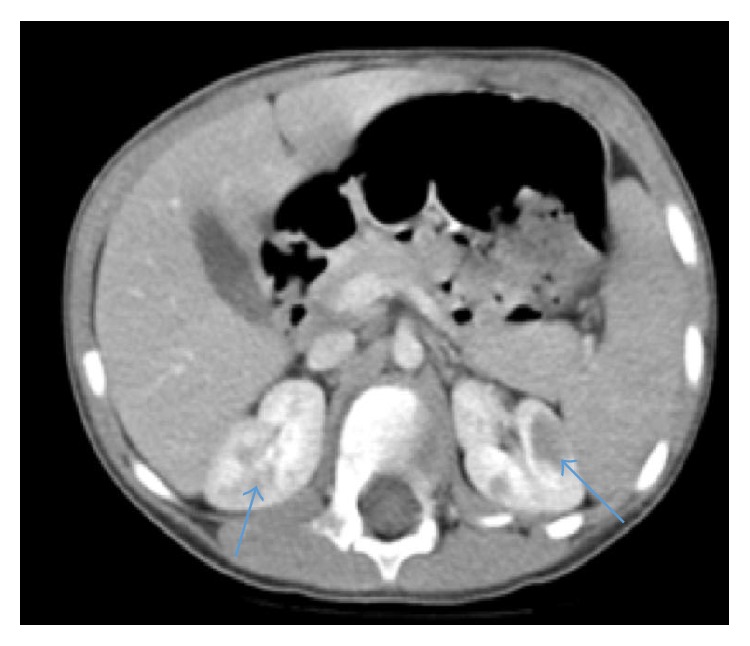
CT abdomen with contrast on initial presentation. Arrows showing bilateral hypodense lesions represent leukemic deposits.

A four-year-old boy with unremarkable past medical history was seen in the emergency department (ED) with low-grade fever and abdominal pain of a few days duration. A complete blood count (CBC) and abdominal computed tomography (CT) study were performed for evaluation for possible appendicitis. The CBC showed a white blood cell (WBC) count of 8.1 × 10^9^/L with a differential of 22% neutrophils, 68.9% lymphocytes, and an absolute neutrophil count of 1.8 × 10^9^/L, hemoglobin of 13.3 g/dL, and platelets of 170 × 10^9^/L. Chemistry studies showed blood urea nitrogen of 2.5 mmol/L and creatinine of 33.5 *μ*mol/L. Urinalysis was normal. Although the patient was ultimately diagnosed with viral gastritis and sent home with supportive treatment, CT imaging revealed bilateral renal enlargement with multiple small subcortical and cortical medullary lesions that were hypoenhancing and were determined to be consistent with nephrogenic rests per reading by a pediatric radiologist (Figure 1). In the absence of other significant clinical findings, the renal abnormalities were interpreted as nephroblastomatosis, and the patient was referred to a pediatric nephrology clinic for further follow-up and surveillance for Wilms' tumor transformation. At follow-up, repeat urinalysis was again within normal limits and urine culture was negative. Because of the concern for malignancy such as lymphoma, which can present with similar radiological findings, the need for renal biopsy was discussed with the pediatric oncologist and the pediatric surgeon. However, given the risk versus benefit assessment, close radiologic follow-up was chosen as the management plan instead of biopsy.

Fourteen days after initial presentation, the patient again complained of abdominal pain accompanied by a low-grade fever. Repeat CBC showed WBC of 3.5 × 10^9^/L with a differential of 6% neutrophils, 85% lymphocytes, and an absolute neutrophil count of 351 × 10^9^/L, hemoglobin of 11.1 g/dL, and platelet count of 21 × 10^9^/L. The erythrocyte sedimentation rate was 71 mm/hr. The pediatric oncology service was consulted for evaluation for malignancy. Peripheral blood smear was remarkable for the presence of abundant immature lymphoid cells representing 90% of the total white cells. Bone marrow aspirate and biopsy flow cytometry and cytogenetic studies were conclusive for early B cell leukemia.

**Figure 2:**
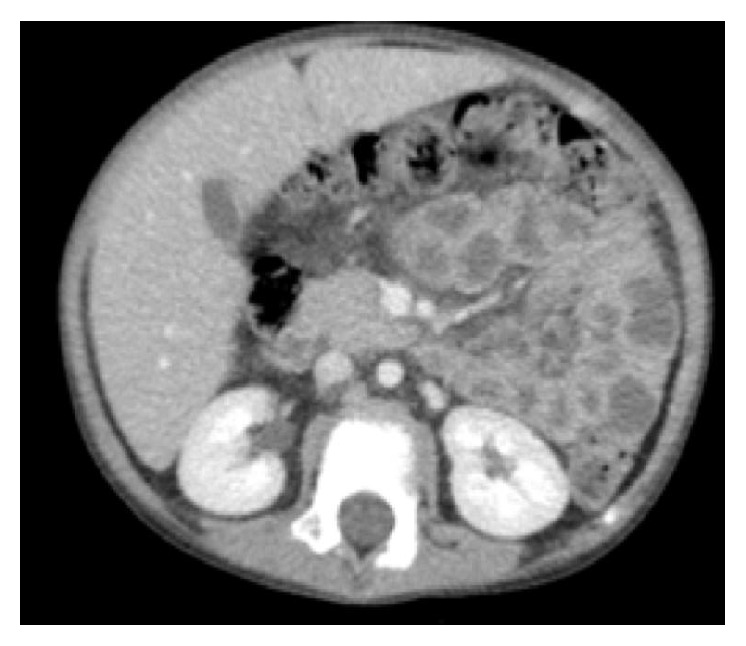
CT abdomen with contrast after induction therapy for ALL. Interval resolution of small hypodense subcortical lesions within both kidneys, consistent with favorable response to therapy of leukemic deposits in the kidney.

Treatment was initiated following the guidelines of COG AALL0932. After one week of induction chemotherapy, repeat CT study showed complete interval resolution of all renal hypodensities (Figure 2).

## 3. Discussion

Renal enlargement in childhood is associated with a broad differential diagnosis including congenital anomalies, hydronephrosis, infection, and malignancy [3]. Leukemic infiltration in the kidney is common in the later stages of ALL in children [4]. However, isolated renal involvement is rare as an initial finding of ALL [3–5]. This patient had significant kidney lesions despite a completely normal initial work-up at the time of the initial presentation. Thus the initial differential diagnosis by the ED physician did not include malignancy. Moreover, the patient had nonspecific abdominal signs and symptoms with normal urinalysis and negative urine culture making urinary tract infection unlikely. A more complete list of differential diagnoses for similar lesions may include lymphoma, nephroblastomatosis, simple cysts, angiomyolipoma, and metastases in addition to renal leukemic involvement [6]. In the absence of classical signs and symptoms of leukemia, and with the only renal abnormalities on CT imaging, a diagnosis for this patient was elusive.

ALL infiltrates may vary on CT imaging. They may present as enlargement of the kidneys, either unilateral or bilateral, or as low-attenuation focal parenchymal lesions, either unilateral or bilateral and either solitary or multiple [6]. These focal lesions can be difficult to distinguish from nephroblastomatosis, which has a similar radiological picture [6, 7]. In this case, CT imaging showed bilateral renal enlargement with multiple small subcortical and cortical medullary lesions that were hypoenhancing and overall more consistent with nephrogenic rests. Nephroblastomatosis is multifocal or diffuse nephrogenic rests which are defined as persistent foci of embryonic cells beyond 36 weeks' gestation and have the potential to transform into Wilms' tumor [7]. Therefore, this patient was closely followed in the nephrology clinic with repeat imaging studies every 3 months for monitoring for transformation.

In most cases, features of classic leukemia at initial presentation differentiate renal involvement in leukemia from nephroblastomatosis. However, in this case, these classic findings were absent leading the team to conclude that the CT findings were most suggestive of nephroblastomatosis. Although no cytology or histology studies from the lesion were performed, their complete resolution shortly after initiation of ALL specific chemotherapy demonstrates that the lesions were in fact leukemic infiltrates after all.

In conclusion, this case demonstrates that renal involvement may be the initial finding of early stage of ALL. With this in mind, children with lesions resembling nephroblastic rests need to be followed up closely in the pediatric nephrology clinic.
